# Device–device interference triggered by an abandoned pacemaker: a case report

**DOI:** 10.1093/ehjcr/ytae595

**Published:** 2024-11-05

**Authors:** Dong Wang, Johanna Mueller-Leisse, Henrike A K Hillmann, Jörg Eiringhaus, David Duncker

**Affiliations:** Hannover Heart Rhythm Center, Department of Cardiology and Angiology, Hannover Medical School, Carl-Neuberg-Straße 1, Hannover 30625, Germany; Hannover Heart Rhythm Center, Department of Cardiology and Angiology, Hannover Medical School, Carl-Neuberg-Straße 1, Hannover 30625, Germany; Hannover Heart Rhythm Center, Department of Cardiology and Angiology, Hannover Medical School, Carl-Neuberg-Straße 1, Hannover 30625, Germany; Hannover Heart Rhythm Center, Department of Cardiology and Angiology, Hannover Medical School, Carl-Neuberg-Straße 1, Hannover 30625, Germany; Hannover Heart Rhythm Center, Department of Cardiology and Angiology, Hannover Medical School, Carl-Neuberg-Straße 1, Hannover 30625, Germany

**Keywords:** Device interference, Cardiac implantable electronic device, Pacemaker, Pacemaker mode, Recommended replacement time, Case report

## Abstract

**Background:**

Cardiac implantable electronic devices (CIEDs) are prone to electromagnetic interference. Common sources include household electronics and industrial machinery. However, medical equipment can also trigger interferences and cause CIED malfunction.

**Case summary:**

We report on a 79-year-old male with sudden onset of presyncope. He has a long history of device therapy, including an active VVI leadless pacemaker and an abandoned abdominal pacemaker with epicardial leads. Automatic reactivation of the abandoned pacemaker due to reaching end-of-life mode led to interaction with the active pacemaker, inhibiting it in its function. Due to elevated capture threshold and insufficient output, pacing by the reactivated pacemaker was accompanied by intermittent loss of myocardial capture and patient symptoms. By changing the mode to VOO and increasing the pacing rate of the active pacemaker, the interaction was prevented as an intermittent solution. The final therapy consisted of explanting the pulse generator of the abdominal pacemaker.

**Discussion:**

We present a patient with a deactivated abandoned cardiac pacemaker, which self-activated after reaching end-of-life mode and triggered an interaction with his active pacemaker. This case emphasizes the importance of explanting old devices to avoid potential interaction.

**Learning points**

- Deactivated cardiac implantable electronic devices in OOO mode can reactivate themselves and start pacing again.
- Explantation of abandoned devices should be considered even in old patients to avoid potential interaction with other devices.

## Introduction

In recent years, the number of patients that have been fitted with cardiac implantable electronic devices (CIEDs) such as cardiac pacemakers or implantable cardioverter defibrillators (ICDs) has strongly increased.^1^

Electromagnetic interference (EMI) can occur as a result of exposure of a CIED to an electromagnetic signal from other devices such as a smartphone, metal detector, headphones, household appliances, or industrial machinery. In rare cases, internal and external medical devices, as well as surgical procedures, can also cause EMI and CIED malfunction.^2,3^ Electromagnetic interference can be interpreted by pacemaker as an intrinsic cardiac signal and result in inhibition of pacing, leading to bradycardia and potentially cardiac arrest. Similarly, EMI in patients with ICD can lead to inappropriate shocks due to perceived ventricular tachyarrhythmia.^4^

In this case report, we present a pacemaker patient who suffered from bradycardia due to reactivation and subsequent interference of his abandoned pacemaker.

## Summary figure

**Figure:**
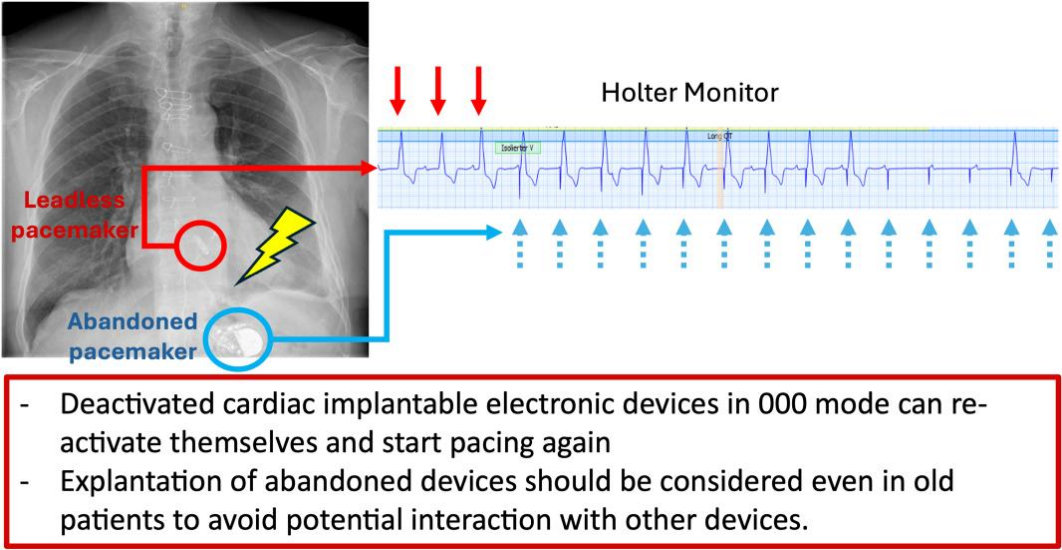


## Case presentation

A 79-year-old male patient presented in the emergency department with intermittent dizziness and presyncope. The complaints started suddenly on the day of the presentation. At presentation, he had a baseline blood pressure of 112/65 mmHg, a heart rate of 80 b.p.m. and an extensive cardiovascular and device history.

His cardiovascular history included a coronary artery disease with a condition after coronary bypass surgery and two non-ST-elevation myocardial infarction. Furthermore, the patient suffered from paroxysmal atrial fibrillation and had a CHA_2_DS_2_-VASc score of 5 points. His medication included bisoprolol, ramipril, apixaban, torsemide, atorvastatin, ezetimibe, and pantoprazole.

**Figure 1:**
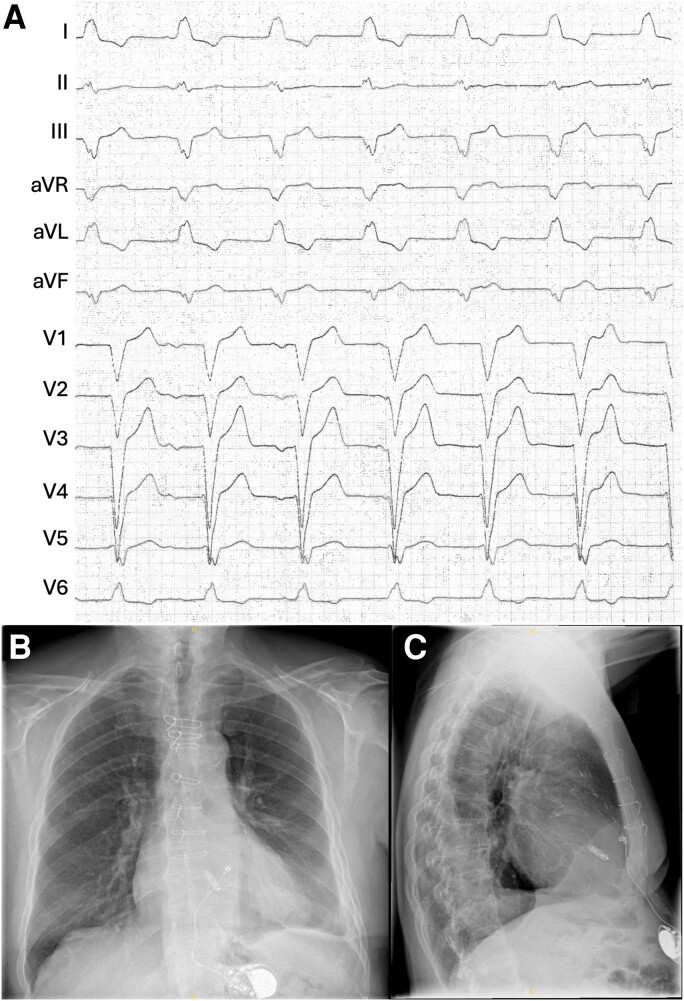
Twelve-lead electrocardiogram (*A*) and chest X-ray in posteroanterior (*B*) and lateral (*C*) view of the patient at initial presentation in the emergency department. Both leadless pacemaker and abdominal pacemaker are visible in the chest X-ray.

No abnormalities were found in the physical examination. An initial 12-lead electrocardiogram (ECG) revealed a pacemaker-stimulated rhythm with a frequency of 80 b.p.m. (*Figure 1A*). The patient had a known medical history for device therapy: At the age of 68, he developed a complete atrioventricular block, which required the implantation of a transvenous dual chamber pacemaker. Seven years later, this system was explanted due to infection of the pacemaker leads. A single-chamber pacemaker with epicardial electrode was implanted in the abdominal position. The abdominal pacemaker revealed suboptimal electrode values with a high capture threshold and low sensing values and reached the elective replacement indicator after only 5 years. The patient did not have a sufficient intrinsic rhythm and was pacemaker dependent. Instead of revising the epicardial lead, the decision was taken to implant a leadless pacemaker. As the patient refused an additional surgery for the explantation of the abdominal device at that moment, it was turned to OOO mode and remained in the patient.

A chest X-ray confirmed the presence of the leadless pacemaker and abandoned abdominal pacemaker but did not show abnormal findings (*Figure 1B* and *C*). Echocardiography revealed a normal left and right ventricular ejection fraction. Laboratory examination revealed a mild anaemia and the previously known reduced kidney function.

**Figure 2:**
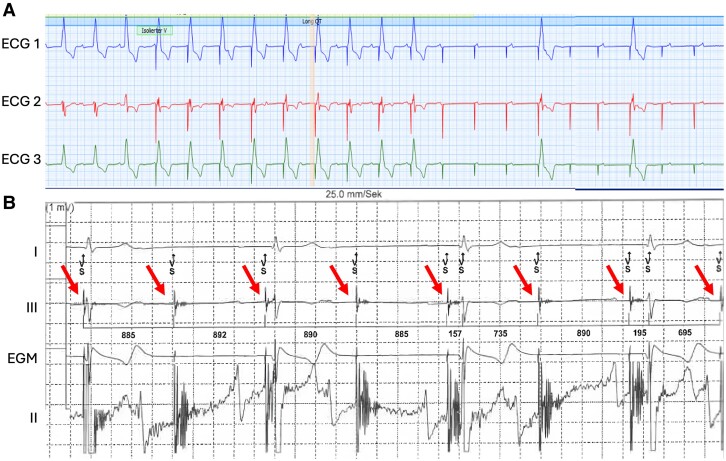
Symptomatic episodes captured by the Holter monitor (*A*) and leadless pacemaker (*B*). (*A*) Holter monitor shows different stimulation spikes with intermittent loss of capture. (*B*) Interrogation of the leadless pacemaker depicts interference with external stimulation signals (diagonal red arrows) that were classified as sensing signals.

The patient was monitored via Holter. On the next day, the patient experienced dizziness again. The Holter monitor recorded a heart rate of 30 b.p.m. Detailed examination of bradycardic episodes showed QRS complexes with different stimulation spikes and stimulations without subsequent QRS complexes (*Figure 2A*). Interrogation of the leadless pacemaker presented episodes depicted in *Figure 2B*. Interference signals were classified as sensing signals by the leadless pacemaker and inhibited stimulation (diagonal red arrows). The leadless pacemaker was in VVIR mode at that time.

**Figure 3:**
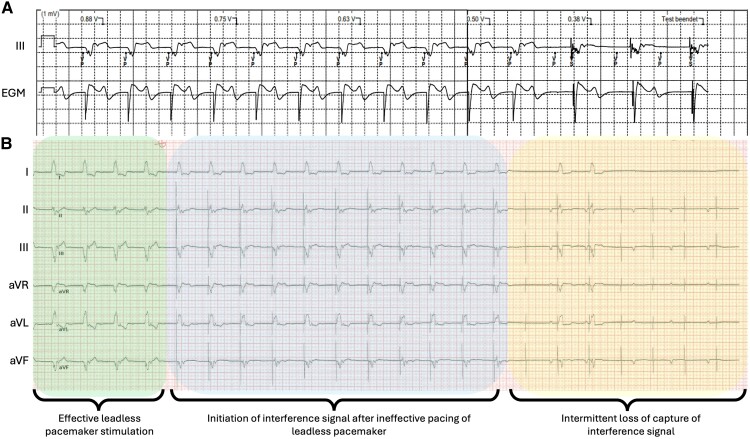
Leadless pacemaker stimulation with decreasing output recorded on the device programmer (*A*) and external electrocardiogram (*B*). Effective pacing at high output is shown in the left section. Ineffective pacing of the leadless pacemaker is associated with initiation of an interference signal (middle section). Interference signal is associated with intermittent loss of capture (right section).

To better understand the underlying mechanism, leadless pacemaker stimulation with decreasing output was performed (*Figure 3A*). At high output, stimulation triggered an appropriate QRS response (*Figure 3B*, left section). However, when stimulating below the pacing threshold, loss of capture was accompanied by the appearance of another regular stimulation spike (*Figure 3B*, middle section). This stimulation was associated with intermittent loss of capture and clinical complaints of the patient (*Figure 3B*, right section).

**Figure 4:**
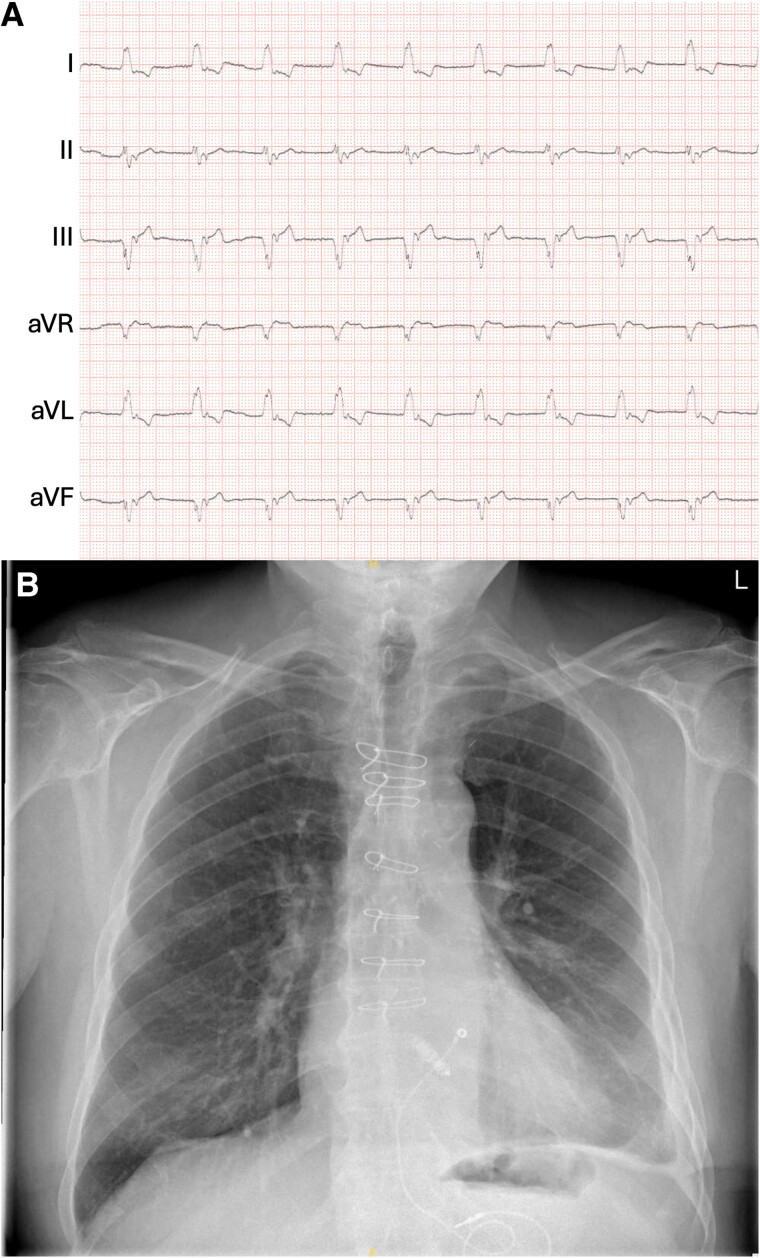
Electrocardiogram after programming the leadless pacemaker to VOO mode (*A*). Chest X-ray after the explantation of the abdominal pulse generator (*B*).

The origin of the interference signal was the abandoned abdominal pacemaker, which had turned to VVI mode after reaching end-of-life (EOL) mode. Due to the previously known high pacing threshold, this resulted in intermittent loss of capture from the abdominal device, but still inhibiting the leadless device resulting in intermittent asystole. Interrogation or reprogramming of the abdominal device was not possible in the status of EOL. Therefore, the leadless pacemaker was temporarily programmed to VOO mode with an intervention frequency above the abandoned abdominal pacemaker to prevent loss of capture as an immediate intervention (*Figure 4A*). Final therapy resulted in the explantation of the abdominal pulse generator (*Figure 4B*). The patient was discharged without further complaints.

## Discussion

Cardiac implantable electronic devices are complex medical devices, and detailed knowledge of their behaviour is critical for health care providers managing pacemaker patients.

Modern CIEDs are set to change their behaviour when approaching battery depletion. This process is usually divided into two stages, recommended replacement time (RRT) and EOL. The specific type of automatic reprogramming at replacement notification alert differs between manufacturers or even between pacemaker models. In the majority of devices, reprogramming includes changes in both pacing mode and pacing rate.^5^ Nonetheless in which mode a pacemaker was programmed, once upon reaching RRT, it will turn to its RRT mode with its set pacing mode and rate. This also includes deactivated pacemakers in OOO mode, which can change to an active pacing mode and self-reactivate, when reaching RRT or EOL. While cardiac resynchronization therapy (CRT) pacemakers behave similarly to other pacemakers at RRT, ICDs do not change pacing parameters at RRT, and anti-tachycardiac therapies remain enabled.^6^

Automatic reprogramming, including self-activation, can also occur during electrical reset or magnet operation.^5^ Electrical reset can be triggered by exposure to EMI, such as electrocautery and defibrillation, and causes initiation of a ‘back-up mode’, which for safety reasons is an active mode. This mode usually involves a high-output VVI pacing at a fixed pacing rate.^5^ Same applies to the magnetic mode, when a magnet is placed near the device, pacing mode changes from the programmed mode to DOO, VOO, or AOO, and a fixed device-specific pacing rate.

Automatic changes in program settings during battery depletion can lead to patient discomfort and symptoms.^7–9^ The most common underlying mechanism is loss of atrioventricular (AV) synchrony, followed by loss of rate response. Symptoms include dyspnoea, fatigue, oedema, presyncope, palpitations, chest pain, and syncope.^7^

In a study evaluating patients with two CIEDs *in situ*, two cases of symptomatic device interaction after RRT-induced self-activation were described.^6^ In one patient, self-activated pacing of the abandoned device caused interference with his active CRT device and led to significant reduction of biventricular pacing, requiring explantation of the abandoned device. In the second patient, the abandoned device displayed unipolar ventricular lead capture, which required the new device to be programmed at a rate greater than the abandoned device.^6^ In general, the existence of two CIEDs in one patient is rare and limited to individual cases.^6,10^

The 2021 guidelines of the European Society of Cardiology and 2018 guidelines of the American College of Cardiology/American Heart Association provide management options for a pacemaker that is no longer indicated.^11,12^ The first option, which is leaving the pacemaker generator and leads *in situ*, is the preferred approach for selected old and frail patients. The second option is explanting the pacemaker generator and abandoned leads. This comes with a low procedural risk, but abandoned leads can be associated with future infections and prohibit magnetic resonance imaging (MRI) examination. The last option is explanting the complete system of both pacemaker generator and leads. This option comes with the highest procedural risks but eliminates all possible future device-related complications. The guidelines do not state which option is the preferred one but recommend a patient-centred approach, in which the decision should be made by the patient together with his doctor.

In conclusion, this case report emphasizes that serious morbidity can arise from abandoned CIEDs left *in situ*. A deactivated CIED can reactivate itself and cause interaction with medical electrical devices. Explantation of abandoned devices should be considered even in old patients to avoid potential interaction.

## Data Availability

The data underlying this article are available in the article and upon contacting the corresponding author.

## Lead author biography

Dr Dong Wang obtained his medical degree from the University of Hamburg, Germany and underwent his postdoctoral training at the University of California, San Francisco, USA. He specializes in cardiology at Hannover Medical School, Germany and has a special interest in heart failure and cardiac electrophysiology.

**Consent:** The authors have received informed consent from the patient to publish this anonymized case report, in accordance with the COPE guidelines.

**Funding:** The authors did not receive any funding for this project.
